# Healthcare providers’ experiences in caring for MDRO carriers with a focus on the moral dimensions of care: a systematic review

**DOI:** 10.1093/jac/dkag059

**Published:** 2026-03-16

**Authors:** Carlijn Damsté, Hester Stoorvogel, Anke Oerlemans, Marjan Knippenberg, Jelle van Gurp, Jaap Ten Oever, Marlies Hulscher

**Affiliations:** IQ Health Science Department, Radboud University Medical Center, Nijmegen, The Netherlands; Radboud Community for Infectious Diseases (RCI), Radboud University Medical Centre, Nijmegen, The Netherlands; IQ Health Science Department, Radboud University Medical Center, Nijmegen, The Netherlands; Radboud Community for Infectious Diseases (RCI), Radboud University Medical Centre, Nijmegen, The Netherlands; Department Internal Medicine, Radboud University Medical Centre, Nijmegen, The Netherlands; IQ Health Science Department, Radboud University Medical Center, Nijmegen, The Netherlands; IQ Health Science Department, Radboud University Medical Center, Nijmegen, The Netherlands; IQ Health Science Department, Radboud University Medical Center, Nijmegen, The Netherlands; Radboud Community for Infectious Diseases (RCI), Radboud University Medical Centre, Nijmegen, The Netherlands; Department Internal Medicine, Radboud University Medical Centre, Nijmegen, The Netherlands; IQ Health Science Department, Radboud University Medical Center, Nijmegen, The Netherlands; Radboud Community for Infectious Diseases (RCI), Radboud University Medical Centre, Nijmegen, The Netherlands

## Abstract

**Objectives:**

Providing care to multi-drug resistant organisms (MDRO) carriers has a profound impact on healthcare providers (HCPs), potentially challenging their personal and professional values. Understanding HCPs’ experiences is key to addressing challenges in providing care to carriers and to support HCPs when providing this care. This review aims to examine experiences of HCPs with a focus on moral dimensions of care.

**Methods:**

We systematically searched Cochrane library, CINAHL, EMBASE, PsycINFO, PubMed and Web of Science. Experiences were analysed using a thematic analysis approach. This review was registered in PROSPERO (CRD42023418340).

**Results:**

Eighteen studies were included, primarily conducted in hospitals and nursing homes. Experiences were categorized into three levels: the individual HCP, the care practice and the institutional setting. First, HCPs are uncertain about personal health and safety, have different knowledge levels and question proportionality of infection prevention and control (IPC) measures. Second, HCPs say that wearing PPE limits connection with carriers and note various other challenges regarding the care relationship such as considering how often to enter an isolation room. Third, HCPs face shortages in resources such as time, experience poor infrastructure making it difficult to adhere to IPC measures and specify a need for management support regarding caring for carriers. Moral dimensions of care received limited attention. An example is that HCPs do not want to betray colleagues who ignore IPC measures, but simultaneously fear transmission due to poor compliance.

**Conclusions:**

The diverse experiences provide input for future interventions to support HCPs in caring for MDRO carriers. The limited attention to moral dimensions of care restricts understanding of HCPs’ values and challenges. Strengthening support requires further research, particularly qualitative studies led by ethics experts.

## Introduction

Antimicrobial resistance (AMR) is a serious global health threat.^1,2^ In healthcare settings, infection prevention and control (IPC) measures including contact precautions are applied to prevent transmission of multi-drug resistant organisms (MDROs).^3,4^

Research has shown that healthcare providers (HCPs) experience adverse effects when providing care to MDRO carriers, such as fear of transmission and stress.^5–7^ Concurrently, MDRO carriers are negatively affected by IPC measures designed to limit the spread of MDROs. They often experience stress and a decline in their mental well-being.^8,9^ While a systematic review of MDRO carriers’ experiences has been conducted,^8^ a comprehensive overview of HCPs’ experiences is still lacking.

Providing care to MDRO carriers can be challenging and may place HCP’s personal and professional values under pressure. For instance, HCPs must balance the responsibility to protect vulnerable individuals by preventing transmission with the obligation to ensure the well-being of the carrier.^10^ Continual pressure on personal and professional values may lead to moral distress, which can result in various biological, psychological and stress-related responses, such as burn-out.^11,12^ Addressing these challenges is particularly important given the widely recognized constraints of time scarcity and workforce shortages in healthcare, which have been identified as urgent priorities for action.^13^ Despite this, limited insight exists into the moral dimensions of care experienced by HCPs.^6,14,15^

Therefore, this review examines the experiences of HCPs across countries and healthcare settings, with a focus on the moral dimensions of care. These moral experiences pertain to the HCP’s sense ‘that values that they deem important are being realized or thwarted in care’.^16^ Understanding HCPs’ experiences is crucial to address the challenges in providing care to MDRO carriers and to support HCPs when providing this care.

## Methods

We performed a systematic literature review, registered in PROSPERO (ID = CRD42023418340). Adaptations of the protocol are presented in Supplementary material S1 (available as Supplementary data at *JAC* Online). We reported in accordance with the PRISMA 2020 statement (see Table S1).^17^

### Search strategy and databases

We developed a search strategy with the help of a librarian by combining keywords and synonyms for ‘healthcare providers’, ‘carriers of antimicrobial resistant organisms’, ‘care’ and ‘experiences’ (see Table S2). We searched Cochrane library, CINAHL, EMBASE, PsycINFO, PubMed and Web of Science for relevant articles. We conducted the initial search on 28 February 2023 and updated this search on 30 January 2025. We performed a backward citation search among included articles to identify additional relevant reviews.^18^

### Eligibility criteria

Studies were included if they (i) were peer-reviewed articles containing original data, and (ii) described experiences in providing care to MDRO carriers from the perspective of a healthcare provider. We defined as experiences thoughts and feelings of HCPs while providing care to carriers, and of how this affected their actions. There were no restrictions on publication date. Details of inclusion and exclusion criteria are provided in Table S3.

### Study selection

Duplicate studies were removed in Endnote (version 21, Clarivate, PA, USA).^19^ Initial screening was conducted based on title and abstract using Covidence systematic review software (Veritas Health Innovation, Melbourne, Australia). For studies considered relevant, full texts were assessed. During initial screening (including the updated search), 1490 of 6545 (23%) were independently reviewed by two researchers (C.D., M.K.). During full-text screening (including the updated search), 39 of 42 (93%) were independently reviewed by two researchers (C.D., M.H.). Remaining studies were reviewed by C.D. Discrepancies and doubts were discussed until consensus was reached, and if necessary, a third team member (A.O., J.v.G. or J.t.O.) was consulted.

### Data collection and synthesis

Key characteristics were extracted from the studies. Original data from the studies were coded using a thematic analysis approach,^20^ using ATLAS.ti (version 24.0.0, Scientific Software Development, GmbH, Berlin, Germany). Two researchers (C.D., H.S.) independently conducted open coding for 10/18 (56%) of the included studies. Each study was coded independently and subsequently discussed together before proceeding to the next. Experiences were coded, with a particular focus on the moral dimensions of care. Inspired by Hunt *et al.*,^16^ we defined moral experiences as the HCP’s sense ‘that values that they deem important are being realized or thwarted in care’. After coding 10 studies, only minimal discrepancies remained, indicating an aligned interpretation of the data. Therefore, the remaining studies were coded by C.D. and reviewed by H.S. Any discrepancies or doubts were discussed. Finally, the research team constructed subthemes and themes from the assigned codes in three sessions.

### Quality assessment

We used the Mixed Methods Appraisal Tool to assess the quality of included studies (see Table S4).^21^ Ten out of 18 studies (56%) were independently assessed by two researchers (C.D., H.S.). C.D. assessed the remaining studies, after which H.S. reviewed the assessment. Any discrepancies were discussed. No study was excluded based on quality.

## Results

### Study selection

**Figure 1.**
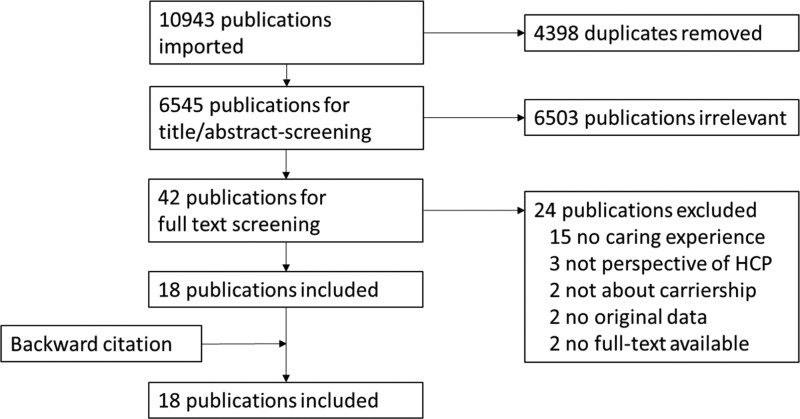
PRISMA flowchart of study selection.

The search strategy yielded 6545 articles. After title/abstract screening, we assessed 42 full-text articles for eligibility, which led to the inclusion of 18 studies (Figure 1). For a list of the excluded studies, see Table S5.

### Study characteristics

Key characteristics for each study are provided in Table 1. Studies were either qualitative (*n* = 12; 67%) or quantitative descriptive studies (*n* = 6; 33%). Publication dates ranged from 2002 to 2024 (median = 2018, 25th percentile = 2014, 75th percentile = 2022). Studies took place in nine countries. Study settings included mainly hospitals (*n* = 15) and nursing homes (*n* = 3). The targeted types of HCPs included mostly nurses (*n* = 18) and physicians (*n* = 10). HCPs provided care to patients with different types of MDRO colonization, although in 11 studies it was not clear whether the IPC measures were applied for colonization or infection. Types of IPC measures were poorly specified. The assessed quality of the studies varied (see Table S4). Five studies were assessed as moderate quality, meeting 40%–80% of the MMAT criteria.^24,27,32,34,35^ The remaining 13 studies were assessed as high-quality (≥80% of MMAT criteria met).^5–7,22,23,25,26,28–31,33,36^

**Table 1.** Key characteristics per study

Author (year)	Country	Study design and methods	Setting	Study population (type of HCPs)	Sample size	Type of MDRO
Qualitative studies						
Andersson *et al.* (2016)^5^	Sweden	Qualitative study with semi-structured individual interviews	Hospitals, nursing homes	Nurses, nursing assistants	15	MRSA; unclear colonization and/or infection
Eli *et al.* (2020)^22^	Israel	Qualitative study with semi-structured individual interviews	Large tertiary hospital	Physicians, nurses, nursing assistants, cleaning staff^a^	15	CPE; unclear colonization and/or infection
Harris *et al.* (2023)^6^	Australia	Qualitative study with semi-structured individual interviews	Public health organization, HCPs work in hospital	Physicians, nurses, allied health professionals^a^	24	Different MDROs; colonization
Herbst *et al.* (2019)^23^	Germany	Qualitative study with semi-structured focus groups	University clinic, large community hospital	Physicians, nurses, psychologists, pastoral caregivers, social workers, allied health professionals	19	Different MDROs; unclear colonization and/or infection
Kaba *et al.* (2017)^24^	Canada	Qualitative study with semi-structured individual interviews	Large community hospital	Nurses^a^	14	MRSA, VRE; unclear colonization and/or infection
Langeveld *et al.* (2024)^25^	The Netherlands	Qualitative study with semi-structured focus groups	Different organizations providing home-based nursing care	Nurses, nursing assistants	34	Different MDROs; colonization
Lindberg *et al.* (2014)^26^	Sweden	Qualitative study with semi-structured focus groups	Hospital, primary care settings	Physicians, nurses^a^	12	MRSA; colonization
Mitchell *et al.* (2002)^27^	Australia	Qualitative study with structured individual surveys	Urban hospital	Nurses	11	VRE; unclear colonization and/or infection
O’Connor *et al.* (2023)^28^	Ireland	Qualitative study with semi-structured individual interviews	Large acute hospital	Nurses, healthcare attendants, allied health professionals	11	CPE; colonization
Seibert *et al.* (2014)^29^	USA	Qualitative study with semi-structured individual interviews	Hospital with acute healthcare settings	Physicians, nurses, allied health professionals^a^	26	MRSA; unclear colonization and/or infection
Tiedtke *et al.* (2018)^30^	Germany	Qualitative study with semi-structured individual interviews	2 hospitals	Physicians, nurses, social workers, psychologists, music therapists, allied health professionals, volunteer assistants without training in care, cleaning staff^a^	35	Different MDROs; unclear colonization and/or infection
Wiklund *et al.* (2015)^31^	Sweden	Qualitative study with semi-structured individual interviews	Hospital with acute healthcare settings, nursing homes	Physicians, nurses, nursing assistants	23	ESBL; unclear colonization and/or infection
Quantitative descriptive studies						
DaSilva *et al.* (2010)^32^	Brazil	Survey	State large hospital	Nurses, nursing assistants	318	MRSA; colonization
Harris *et al.* (2020)^33^	Australia	Survey	Public health organization covering 8 inpatient facilities and variety of community-based services	Nurses	53	Different MDROs; colonization
Khan *et al.* (2006)^34^	USA	Survey	Tertiary care centre	Physicians, nurses	155	Different MDROs; unclear colonization and/or infection
Krein *et al.* (2020)^35^	USA	Survey	Tertiary medical care centre	Physicians, nurses, allied health professionals	263	MRSA; unclear colonization and/or infection
Langeveld *et al.* (2022)^7^	The Netherlands	Survey	Hospitals, home healthcare, nursing homes, general practice, mental healthcare, disability care	Nurses, nursing assistants, assistants without training in care, social workers, other	974	Different MDROs; colonization
Watson *et al.* (2023)^36^	Australia	Survey	University hospital	Physicians, nurses, allied health professionals^a^	105	CPE; unclear colonization and/or infection

^a^This study incorporated additional perspectives beyond those discussed here; however, these perspectives were excluded from the analysis, as they did not involve HCPs with direct care contact with carriers.

### Experiences of HCPs with providing care to MDRO carriers

**Figure 2.**
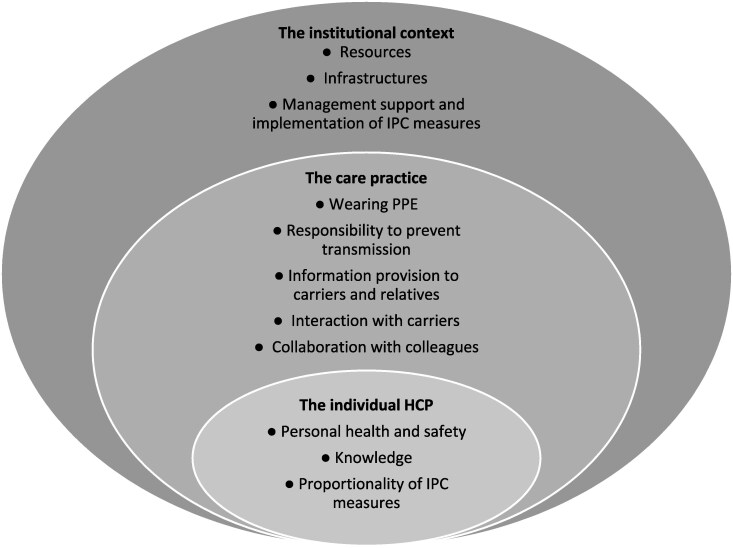
Three levels with subthemes illustrating the experiences of HCPs when providing care to MDRO carriers. The experiences manifest across these three levels: (i) the intrapersonal perspectives of HCPs (the individual HCP), (ii) the care practices and belonging interactions with patients, relatives and colleagues in which HCPs operate (the care practice) and (iii) the organizational and institutional settings that shape the work of HCPs (the institutional context).

Findings are presented into three levels regarding (i) the individual HCP, (ii) the care practice and (iii) the institutional setting (Figure 2). When studies describe how these experiences affect caregiving, this is also reported. Examples of descriptions of moral dimensions of care are shown per level (Table 2–4).

**Table 2.** Examples of moral dimensions of care: the individual HCP

Subtheme	Example of moral dimension	Quote	Study
Proportionality of IPC measures	Doubts about the proportionality of IPC measures	‘Safety first and at the same time we have to think about how safe we want it. I mean, if the actual risk is so incredibly low, does everyone have [to] protect themselves from that?’ (table 2, quote 1,^25^)	^25^
	Feeling that IPC compromises quality of care	Nurses indicated the cumulative effect of time spent on IPAC compromised the quality and continuity of patient care and were concerned they were not meeting the psychosocial needs of isolated and nonisolated patients. (results section, p. 201,^24^)	^22,24,28,29,33^

Examples of moral dimensions of care are provided. A quote of such an example is shown. Studies that hint to similar descriptions are given.

**Table 3.** Examples of moral dimensions of care: the care practice

Subtheme	Example of moral dimension	Quote	Study
Wearing PPE/interactions with carriers	Feeling unable to provide appropriate patient care (and sometimes therefore frustrated) due to MDRO carriership	As the total number of patients and those in contact isolation under the daily care of the HCW increased, more respondents perceived an inability to efficiently respond to the needs of contact isolation patients. (results section, p. 410,^34^)	^24,28,34^
Responsibility to prevent transmission	Feeling responsible to prevent transmission to others while safeguarding carriers’ well-being	The fear among the participants sometimes caused a lack of respect and empathy for ESBL patients, resulting in implementation of excessive protective measures and avoidance of contact with ESBL patients. Some participants expressed feeling ashamed about their actions. ‘When I came back after my holiday, I went straight to the lady and gave her a hug and a kiss on the forehead. Then my colleague said, ‘No, don’t do that!’, and then I was shocked (…), I thought, what a fool I am! Then I felt really ashamed about what I had done.’ (results section, p. 1306,^31^)	^6,23,30,31^
Interaction with carriers	To a certain extent not wanting to care or different care for a carrier	Fear and insecurity among the participants had a pronounced impact on the nursing care itself. Conflicts arose because no one wanted to care for patients with MRSA or even enter their rooms. (results section, p. 236,^5^)	^5,6,22,28,29,33,34^
Collaboration with colleagues	Not wanting to betray colleagues who ignore IPC measures but simultaneously fearing transmission of MDROs due to poor compliance of colleagues	Participants could sometimes face dilemmas that challenged their ‘loyalty to colleagues’. It was stated that other nurses sometimes did not follow the infection control principles. The participants did not want to ‘snitch’, but, on the other hand, they were afraid that infection could be spread. (results section, p. 238^5^)	^5^

Examples of moral dimensions of care are provided. A quote of such an example is shown. Studies that hint to similar descriptions are given.

**Table 4.** Examples of moral dimensions of care: the institutional setting

Subtheme	Example of moral dimension	Quote	Study
Resources	Hindered IPC compliance and appropriate caregiving due to lack of time, inadequate staffing levels and poor infrastructure	On night shifts, with lower staffing levels, additional staff was needed to make it possible to follow ESBL guidelines, but such help was seldom available. Some managers actively broke the rules by taking too many patients onto the wards (…). A situation with too many patients on the ward would often result in reduced patient safety. ‘What is more important, patient safety and proper care for patients or hospital beds? It’s really sad that they interfere with patient safety.’ (results section, p. 1305^31^)	^24,28,29,31^

Examples of moral dimensions of care are provided. A quote of such an example is shown. Studies that hint to similar descriptions are given.

#### Experiences of the individual HCP and how they affect providing care

##### Personal health and safety

Many studies reported that HCPs experience uncertainty or anxiety regarding the perceived personal risk of MDRO transmission.^5,7,22,23,25,28–36^ HCPs worry about consequences such as becoming sick or losing their job. To cope with the fear of transmission, some HCPs strictly adhere to IPC measures,^31^ or use additional protective measures not recommended by guidelines,^5,23,28,29,31,33^ such as treating a carrier last or using extra gloves. HCPs consider it challenging that MDRO carriership itself is invisible in a patient.^7,25^

By contrast, some HCPs feel sufficiently protected and do not fear transmission while providing care.^5,22,23,28,31,32,34,35^ Some HCPs report that sometimes their fear decreases over time and with experience and knowledge.^26,28,31,33^ Experiences with COVID-19 care have reduced anxiety about MDROs as COVID-19 was perceived as a greater risk among HCPs.^28^

##### Knowledge

Across studies, HCPs report varying levels of knowledge about MDROs, including general information, transmission routes, IPC guidelines, their importance and where to find them, how to wear PPE and how to care for carriers. HCPs experience stress when they are unsure how to apply contact precautions or when worrying about transmission risks of contaminated materials. Ambiguities in IPC protocols can also lead to uncertainty among HCPs.^23,25^ HCPs say that infrequent work or first-time experiences in isolation units contribute to difficulties,^5,22,26,31^ whereas experience in caring for MDRO carriers can make it feel like routine care.^26,31,33^ Some HCPs note that experiences with COVID-19 care have increased awareness and familiarity with IPC measures and PPE usage.^25,28^ Some studies stated that feeling knowledgeable can empower HCPs and vice versa.^25,28,31^ The availability of and willingness to participate in education varies among HCPs.^22,25,29,31,32^

##### Proportionality of IPC measures

HCPs sometimes express doubts about the efficacy or proportionality of IPC measures, such as questioning actual transmission risks.^25^ Some HCPs find PPE cumbersome for small care tasks.^22,29,30^ One study suggested that HCPs’ perceptions of IPC measures as disproportionate could contribute to non-compliance with IPC measures.^25^ Some HCPs feel that IPC measures could compromise quality of care, and they feel constrained by these measures.^22,24,26,28,29,31^ One study—in the context of end of life care—showed that some HCPs prefer personal contact and are willing to find compromises in applying IPC measures, while others prefer strict adherence to prevent transmission to others and themselves.^30^ In addition, some HCPs note that emergency situations could impede proper adherence to IPC measures.^28,29,32^

#### Experiences related to the care practice and how they affect providing care

##### Wearing PPE

HCPs mention that wearing PPE limits contact and connection with carriers.^29,30^ Some note they are able to give sufficient personal attention to carriers,^7^ while others feel unable to respond promptly to the needs of carriers.^24,34^ HCPs note that PPE complicated proper conversations, for example due to a perceived greater distance.^30^ One survey study reported that using PPE sometimes gives rise to feelings of guilt among HCPs.^7^ HCPs experience emotional (e.g. more distance to patient) and/or physical (e.g. heat) discomfort due to wearing PPE.^6,7,25,29,30^

##### Responsibility to prevent transmission

HCPs feel responsible for preventing transmission to other patients or their own family members.^5,7,23,25,27–33,35^ They sometimes feel uncertain about whether they have contributed to transmission, leading to feelings of guilt or worry about potentially spreading MDROs to others.^27,33^ Some explicitly frame the implementation of IPC measures as a moral dilemma between protecting the wider community from transmission and safeguarding the individual well-being of carriers, highlighting the negative impact these measures can have on carriers.^6,23,30,31^

##### Information provision to MDRO carriers and relatives

Explaining carriership and related IPC measures to carriers is often considered challenging by HCPs.^7,25–27,29,31,33^ While some HCPs feel confident in their explanations,^7,27,33^ others worry about upsetting patients with information,^33^ feel unable to explain carriership to patients,^7,25–27,31,33^ or find questions of patients or family members stressful.^25,27,33^ One study mentioned that HCPs sometimes receive critical comments from patients when explaining IPC measures.^7^ Another study highlighted that MDRO carriership is not frequently discussed with patients in care by HCPs.^33^

Several studies also highlighted the importance and challenges of information provision and communication with the relatives of carriers.^25,27,29,31^ Having to inform relatives can also cause HCPs to worry about their reactions. Sometimes, HCPs feel stressed having to answer their questions.^25,27,33^ Some HCPs note reluctance among relatives to follow IPC measures.^27,31^

##### Interaction with carriers

Some studies found that HCPs express empathy for MDRO carriers and felt the need to comfort them,^30,31^ while others perceive the carrier as a threat.^5,31^ Some HCPs prefer not to care for carriers, even taking sick leave instead,^5^ avoiding or fearing entering the isolation room,^5,6,22,29,33,34^ examining them less often,^28,34^ maintaining distance^6^ and avoiding physical contact.^5,22,29,33^ Studies reported fear of transmission as a possible reason for this behaviour. Some HCPs avoid MDRO-marked areas or perceive isolation units as dirty.^22,29,33^ For example, one study reported that an HCP felt punished when assigned to MDRO care.^22^

Other HCPs find MDRO care challenging in a positive way,^5,27^ or consider it just part of their normal work.^29,30^ Several HCPs explicitly mention trying to enter a carrier’s room as often as a non-carrier’s room.^5^ While some HCPs believe there is no difference in care provided to carriers and non-carriers,^5,25,27,34^ others perceive that their own care differs.^34^ Some HCPs feel responsible for providing good patient care and experience frustration when unable to provide appropriate care due to MDRO carriership,^28,30,31^ such as delays caused by additional waiting time. HCPs note that carriers may feel lonely, depressed, imprisoned or overwhelmed by information, implicating that they are aware of the diverse impact of carriership.^5,22,33,34^

##### Collaboration with colleagues

HCPs observe that some colleagues do not adhere to IPC measures or do not feel the need to do so.^25,27,29^ Some studies suggested that this non-adherence could challenge team dynamics.^25,27,32^ While some HCPs accept suboptimal compliance by their colleagues, others face a dilemma between not wanting to betray colleagues who ignore IPC measures and fearing transmission of MDROs due to poor compliance.^5^ Several HCPs feel reluctant to speak up about recent IPC protocols towards colleagues.^5,31^ Others feel responsible for educating colleagues.^5,25,27,29,31^ Some HCPs mention that the entire team should take responsibility for correctly applying IPC measures.^27,29^

One study mentioned that good MDRO care requires cooperation and coordination among co-workers,^23^ for example between those working inside and outside isolation rooms. Another study described that there is reluctance among HCPs to collaborate with nursing colleagues in the isolation unit.^22^ The importance of timely communication and information transfer regarding MDRO status was emphasized,^25,33^ as delays could hinder the implementation of IPC measures. One study—in the context of home-based care—addressed that HCPs experience a lack of colleagues to share their thoughts with, which they found challenging.^25^

Sometimes HCPs feel alone while working.^22,25,27^ Several feel alienated, blamed or stigmatized by colleagues not involved in caring for MDRO carriers,^27^ especially in case of an MDRO outbreak, as they perceive themselves as vectors or gatekeepers of the outbreak. On the other hand, HCPs state that support from their departments and colleagues enhances their sense of connectedness.^27^

#### Experiences related to the institutional setting and how they affect providing care

##### Resources

The availability of resources for MDRO care varies. Time constraints were often mentioned in studies, with MDRO care generally considered more time-consuming and less efficient by HCPs.^5,7,22,23,27–30,33–36^ This is due to the need for MDRO screening, wearing PPE, cleaning materials and answering questions from patients and their relatives about carriership. To save time, HCPs often bundle care tasks,^7,30^ assess care needs first,^30^ or prepare thoroughly before entering an isolation room.^22,23,30^ Studies noted that increased workload and lack of time could result in feeling fatigued^27,28^ or rushed when caring for a carrier,^5,36^ and cutting corners when applying IPC measures.^24,28,29,31^

Many studies mentioned that inadequate staffing levels hinder appropriate caregiving or adherence to IPC measures, contributing to time constraints.^7,27–29,31,32^ In one study, some HCPs emphasize the importance of work planning and assigning MDRO carriers in a way that balances their care with other duties.^29^

Insufficient availability of PPE,^7,32^ cleaning materials^29^ or isolation rooms were also reported.^5,28,29,31^ Some studies noted that MDRO carriers restrict room capacity, sometimes necessitating their transfer to other wards or hospitals.^5,29,31^ In addition, some HCPs recognize that IPC resources could have negative environmental^6^ and financial effects.^6^

##### Infrastructures

HCPs note that isolated areas were not always clearly marked,^22,29^ overcrowded^22,31^ or had poor infrastructure,^28^ which hinders adherence to IPC measures. Issues such as the inappropriate placement of hand hygiene dispensers are specifically mentioned.^28,29^ One study highlighted that visual management boards displaying MDRO carrier data is considered helpful by HCPs.^24^

##### Management support and implementation of IPC measures

HCPs emphasize the need for context-specific and case-specific IPC measures.^23,25,26^ One study stressed that hospital IPC guidelines are not always fully applicable to home-based care.^25^ The same study reported that HCPs in home care feel abandoned by IPC support from hospitals, as it does not acknowledge the context of home-based care.^25^ When IPC measures are not well-suited to the context, this sometimes forces HCPs to find *ad hoc* solutions to prevent transmission.^22,25^

In one study, HCPs suggest that management should advocate for adherence to IPC measures on the work floor.^5^ HCPs experience a lack of support in terms of unavailable IPC guidelines,^5^ no or delayed information on how to care for carriers,^5,25,32^ lack of phlebotomy support in isolation units^28^ and other resource shortages as previously mentioned (see subtheme ‘resources’). Conversely, some HCPs say that their facilities provide adequate knowledge and guidelines.^7,32,33,35^ Guidance from IPC experts is considered helpful by HCPs.^5,22,25,31^

## Discussion

This systematic review including 18 articles is the first that comprehensively summarizes the wide variety of HCPs’ experiences when providing care to MDRO carriers in various healthcare settings. These experiences were categorized into three levels: (i) the individual HCP, (ii) the care practice, and (iii) the institutional setting. While we specifically examined the moral dimensions of care, our analysis revealed that these aspects receive relatively limited attention in the studies reviewed.

We anticipated greater emphasis on the moral dimensions of care in the included studies, given that in challenging working environments, determining and acting on what is the right thing to do is integral to providing care. Our parallel qualitative study has demonstrated that moral tensions are a common occurrence when HCPs are providing care to carriers.^15^ In this review, one example pointing to a moral dimension is that several studies describe situations hinting towards stigmatizing practices,^37^ such as that HCPs experience reluctance to enter an isolation room or view a carrier as a threat. Another example is the sense of responsibility to prevent transmission, indicating the presence of something valuable to protect. Moreover, several articles raised questions about proportionality of IPC policies, reflecting HCPs’ considerations of fairness and justice in relation to adherence to IPC measures, workplace realities and patient well-being.^38^

From infectious diseases outbreaks such as the COVID-19 pandemic, we know that moral dimensions in care are profound and that values can be thwarted in care.^39–41^ Unlike outbreak or pandemic situations, providing care to MDRO carriers is part of routine care and will continue for many years due to the increasing prevalence of AMR. Our parallel qualitative study illustrated that HCPs experience various moral tensions while providing care to carriers.^15^ During the COVID-19 pandemic, support programmes were implemented for HCPs to deal with challenges in care. However, structural support for the challenges HCPs face in routine care for MDRO carriers remains largely absent. We consider it problematic that the included articles show a lack of focus on the moral dimensions of care and the potential presence of moral distress (i.e. when one knows the right thing to do but is challenged to do so),^40^ as such a lack of oversight hinders the ability to understand what HCPs consider valuable and to gain insight into what is truly at stake in routine care practices.^42^ This understanding is necessary to develop adequate support for HCPs. Therefore, we recommend additional research using appropriate methods, such as qualitative studies conducted by researchers with expertise in ethics.

Our findings closely align with existing literature on the experiences of HCPs when providing care to patients with various types of infectious diseases, including variations in knowledge about IPC,^43–45^ fear of transmission^46^ and infrastructure issues.^47^ Hence, it could be proposed that the experiences of HCPs do not necessarily differ between applying IPC measures to MDRO carriers and patients with other infectious diseases. However, from the perspective of MDRO carriers, it is probably different. Patients with acute infections are treated until the infection is resolved, then discharged, often requiring only a single admission. By contrast, carriers are typically admitted to hospitals or reside in healthcare settings for other reasons and are repeatedly confronted with the implications of their carriership, even when they are not actively ill due to the MDRO. Research has shown that MDRO carriers experience a profound negative impact on well-being, including a decline in mental health.^8,9,48^ Given this established negative impact, it is crucial for HCPs to recognize the distinctions between patient categories, even if the nature of care may appear similar from the HCP’s perspective.

Strengths of this systematic review include its methodological rigour and its valuable contribution to the existing body of knowledge by providing an overview of HCPs’ experiences with a focus on moral dimensions of care. Limitations include that we were unable to consider the specific context and prevailing IPC measures, as these were often poorly described in the included studies. In addition, some studies involved HCPs with no experience in caring for MDRO carriers, but we could not account for this due to insufficient data identifying these individuals.

In conclusion, we have highlighted a wide range of HCPs’ experiences when caring for MDRO carriers. These experiences manifest across multiple levels: the personal perspectives they bring, the care practices in which they operate, and the institutional settings that shape their work. Understanding HCPs’ experiences is key to addressing challenges in providing care to carriers and to support HCPs when providing this care. Moral dimensions of care need to be further investigated to better understand the challenges of HCPs while providing care to carriers and being able to develop adequate support for HCPs.

## Supplementary Material

dkag059_Supplementary_Data

## Data statement

The output from the data analysis is available from the corresponding author upon reasonable request.
