# Supplementary material for: Healthcare providers’ experiences in caring for MDRO carriers with a focus on the moral dimensions of care: a systematic review
Source: J Antimicrob Chemother. 2026 Mar 16;81(4):dkag059. doi: 10.1093/jac/dkag059 (PMC13017096; doi:10.1093/jac/dkag059)
Supplement: dkag059_Supplementary_Data [file dkag059_supplementary_data.docx]

**Supplementary data**

**Healthcare providers’ experiences in caring for MDRO carriers with a focus on the moral dimensions of care: a systematic review**

Carlijn Damsté, Hester Stoorvogel, Anke Oerlemans, Marjan Knippenberg, Jelle van Gurp, Jaap ten Oever, Marlies Hulscher

**Index**

- S1: Adaptations to PROSPERO protocol
- Table S1: PRISMA 2020 statement - an updated guideline for reporting systematic reviews
- Table S2: Search strategy
- Table S3: Eligibility criteria
- Table S4: Quality assessed using the Mixed Methods Appraisal Tool
- Table S5: Excluded studies

**S1: Adaptations to PROSPERO protocol**

PROPSPERO ID: CRD42023418340

We further specified our main outcome and adjusted this in PROSPERO with a revision note:

The outcome is experiences. We defined experiences as thoughts and feelings of healthcare providers, and how this impacts their actions. Special attention was paid to moral dimensions of care. Inspired by Hunt *et al.* (2011), we defined moral experiences as the HCP's sense "that values that they deem important are being realized or thwarted in care", including HCP's interpretations of interactions in care that "fall on spectrums of right-wrong, good-bad or just-unjust".

This deviates from our initial formulated outcome. We changed "impact on care" into "experiences". This means that e.g. studies only reporting frequency of visiting carriers, without giving descriptions of thoughts and feelings of healthcare providers, are excluded.

Additionally, we initially planned to use Rayyan software as a screening tool. For practical reasons (familiarity of the screeners with the software), we eventually chose to use Covidence software.

**Table S1: PRISMA 2020 statement - an updated guideline for reporting systematic reviews**

| **Section and Topic** | **Item #** | **Checklist item** | **Reported on page #** |
| --- | --- | --- | --- |
| **TITLE** | | |  |
| Title | 1 | Identify the report as a systematic review. | Page 1 |
| **ABSTRACT** | | |  |
| Abstract | 2 | See the PRISMA 2020 for Abstracts checklist. | Page 2 |
| **INTRODUCTION** | | |  |
| Rationale | 3 | Describe the rationale for the review in the context of existing knowledge. | Page 3 |
| Objectives | 4 | Provide an explicit statement of the objective(s) or question(s) the review addresses. | Page 3 |
| **METHODS** | | |  |
| Eligibility criteria | 5 | Specify the inclusion and exclusion criteria for the review and how studies were grouped for the syntheses. | Page 4, S4 |
| Information sources | 6 | Specify all databases, registers, websites, organizations, reference lists and other sources searched or consulted to identify studies. Specify the date when each source was last searched or consulted. | Page 4 |
| Search strategy | 7 | Present the full search strategies for all databases, registers and websites, including any filters and limits used. | S3 |
| Selection process | 8 | Specify the methods used to decide whether a study met the inclusion criteria of the review, including how many reviewers screened each record and each report retrieved, whether they worked independently, and if applicable, details of automation tools used in the process. | Page 4 |
| Data collection process | 9 | Specify the methods used to collect data from reports, including how many reviewers collected data from each report, whether they worked independently, any processes for obtaining or confirming data from study investigators, and if applicable, details of automation tools used in the process. | Page 4,5 |
| Data items | 10a | List and define all outcomes for which data were sought. Specify whether all results that were compatible with each outcome domain in each study were sought (e.g. for all measures, time points, analyses), and if not, the methods used to decide which results to collect. | Page 4,5  Table 1 |
|  | 10b | List and define all other variables for which data were sought (e.g. participant and intervention characteristics, funding sources). Describe any assumptions made about any missing or unclear information. | Page 4,5  Table 1 |
| Study risk of bias assessment | 11 | Specify the methods used to assess risk of bias in the included studies, including details of the tool(s) used, how many reviewers assessed each study and whether they worked independently, and if applicable, details of automation tools used in the process. | Page 5 |
| Effect measures | 12 | Specify for each outcome the effect measure(s) (e.g. risk ratio, mean difference) used in the synthesis or presentation of results. | n/a |
| Synthesis methods | 13a | Describe the processes used to decide which studies were eligible for each synthesis (e.g. tabulating the study intervention characteristics and comparing against the planned groups for each synthesis (item #5)). | n/a |
|  | 13b | Describe any methods required to prepare the data for presentation or synthesis, such as handling of missing summary statistics, or data conversions. | n/a |
|  | 13c | Describe any methods used to tabulate or visually display results of individual studies and syntheses. | Page 4,5 |
|  | 13d | Describe any methods used to synthesize results and provide a rationale for the choice(s). If meta-analysis was performed, describe the model(s), method(s) to identify the presence and extent of statistical heterogeneity, and software package(s) used. | Page 4,5 |
|  | 13e | Describe any methods used to explore possible causes of heterogeneity among study results (e.g. subgroup analysis, meta-regression). | n/a |
|  | 13f | Describe any sensitivity analyses conducted to assess robustness of the synthesized results. | n/a |
| Reporting bias assessment | 14 | Describe any methods used to assess risk of bias due to missing results in a synthesis (arising from reporting biases). | n/a |
| Certainty assessment | 15 | Describe any methods used to assess certainty (or confidence) in the body of evidence for an outcome. | n/a |
| **RESULTS** | | |  |
| Study selection | 16a | Describe the results of the search and selection process, from the number of records identified in the search to the number of studies included in the review, ideally using a flow diagram. | Page 5,6 |
|  | 16b | Cite studies that might appear to meet the inclusion criteria, but which were excluded, and explain why they were excluded. | n/a |
| Study characteristics | 17 | Cite each included study and present its characteristics. | Table 1 |
| Risk of bias in studies | 18 | Present assessments of risk of bias for each included study. | Page 6, S5 |
| Results of individual studies | 19 | For all outcomes, present, for each study: (a) summary statistics for each group (where appropriate) and (b) an effect estimate and its precision (e.g. confidence/credible interval), ideally using structured tables or plots. | n/a |
| Results of syntheses | 20a | For each synthesis, briefly summarize the characteristics and risk of bias among contributing studies. | n/a |
|  | 20b | Present results of all statistical syntheses conducted. If meta-analysis was done, present for each the summary estimate and its precision (e.g. confidence/credible interval) and measures of statistical heterogeneity. If comparing groups, describe the direction of the effect. | n/a |
|  | 20c | Present results of all investigations of possible causes of heterogeneity among study results. | n/a |
|  | 20d | Present results of all sensitivity analyses conducted to assess the robustness of the synthesized results. | n/a |
| Reporting biases | 21 | Present assessments of risk of bias due to missing results (arising from reporting biases) for each synthesis assessed. | n/a |
| Certainty of evidence | 22 | Present assessments of certainty (or confidence) in the body of evidence for each outcome assessed. | n/a |
| **DISCUSSION** | | |  |
| Discussion | 23a | Provide a general interpretation of the results in the context of other evidence. | Page 13,14 |
|  | 23b | Discuss any limitations of the evidence included in the review. | Page 15 |
|  | 23c | Discuss any limitations of the review processes used. | Page 15 |
|  | 23d | Discuss implications of the results for practice, policy, and future research. | Page 13-15 |
| **OTHER INFORMATION** | | |  |
| Registration and protocol | 24a | Provide registration information for the review, including register name and registration number, or state that the review was not registered. | Page 3 |
|  | 24b | Indicate where the review protocol can be accessed, or state that a protocol was not prepared. | Page 3 |
|  | 24c | Describe and explain any amendments to information provided at registration or in the protocol. | S1 |
| Support | 25 | Describe sources of financial or non-financial support for the review, and the role of the funders or sponsors in the review. | Page 16 |
| Competing interests | 26 | Declare any competing interests of review authors. | Page 16, disclosure forms of authors |
| Availability of data, code and other materials | 27 | Report which of the following are publicly available and where they can be found: template data collection forms; data extracted from included studies; data used for all analyses; analytic code; any other materials used in the review. | Page 16 |

**Table S2: Search strategy**

## Pubmed

(healthcare provider*[tiab] OR health care provider*[tiab] OR health provider*[tiab] OR healthcare professional*[tiab] OR health care professional*[tiab] OR health professional*[tiab] OR healthcare worker*[tiab] OR health care worker*[tiab] OR health worker*[tiab] OR healthcare personnel[tiab] OR health care personnel[tiab] OR health personnel[tiab] OR medical personnel[tiab] OR healthcare practitioner*[tiab] OR health care practitioner*[tiab] OR health practitioner*[tiab] OR medical staff[tiab] OR doctor*[tiab] OR physician*[tiab] OR clinician*[tiab] OR nurs*[tiab] OR paramedic*[tiab] OR therapist*[tiab] OR health personnel[MeSH Terms]) AND (antibiotic resist*[tiab] OR anti-biotic resist*[tiab] OR antibiotics resist*[tiab] OR anti-biotics resist*[tiab] OR antimicrobial drug resist*[tiab] OR anti-microbial drug resist*[tiab] OR antibiotic drug resist*[tiab] OR anti-biotic drug resist*[tiab] OR antibacterial drug resist*[tiab] OR anti-bacterial drug resist*[tiab] OR anti-bacterial resist*[tiab] OR antibacterial resist*[tiab] OR antibiotic non-suscept*[tiab] OR anti-biotic non-suscept*[tiab] OR anti-biotic nonsuscept*[tiab] OR antibiotic nonsuscept*[tiab] OR bacterial drug resist*[tiab] OR bacterial resist*[tiab] OR bacterium resist*[tiab] OR microbial drug resist*[tiab] OR aminoglycoside resist*[tiab] OR daptomycin resist*[tiab] OR fluoroquinolone resist*[tiab] OR macrolide resist*[tiab] OR polymyxin resist*[tiab] OR rifampicin resist*[tiab] OR chloramphenicol resist*[tiab] OR kanamycin resist*[tiab] OR tetracycline resist*[tiab] OR trimethoprim resist*[tiab] OR vancomycin resist*[tiab] OR vancomycine resist*[tiab] OR VRE[tiab] OR carbapenem resist*[tiab] OR CPE[tiab] OR extended spectrum beta lactam*[tiab] OR extended spectrum betalactam*[tiab] OR beta lactam resist*[tiab] OR betalactam resist*[tiab] OR ESBL[tiab] OR MRSA[tiab] OR methicillin resist*[tiab] OR methicilline resist*[tiab] OR multidrug resistant organism*[tiab] OR multi drug resistant organism*[tiab] OR multiple drug resistant organism*[tiab] OR MDRO[tiab] OR multidrug resistant microorganism*[tiab] OR multidrug resistant micro-organism*[tiab] OR multi drug resistant microorganism*[tiab] OR multi drug resistant micro-organism*[tiab] OR multiple drug resistant microorganism*[tiab] OR multiple drug resistant micro-organism*[tiab] OR carrier*[tiab] OR colonised patient*[tiab] OR colonized patient*[tiab] OR colonised individ*[tiab] OR colonized individ*[tiab] OR patient carry*[tiab] OR patients carry*[tiab] OR patient carri*[tiab] OR patients carri*[tiab] OR carrier state[MeSH Terms] OR drug resistance, microbial[MeSH Terms]) AND (care[tiab] OR caring[tiab] OR physician-patient interact*[tiab] OR patient-physician interact*[tiab] OR physician-patient relation*[tiab] OR patient-physician relation*[tiab] OR doctor-patient interact*[tiab] OR patient-doctor interact*[tiab] OR doctor-patient relation*[tiab] OR patient-doctor relation*[tiab] OR nurse-patient interact*[tiab] OR patient-nurse interact*[tiab] OR nurse-patient relation*[tiab] OR patient-nurse relation*[tiab] OR professional-patient relation*[tiab] OR patient-professional relation*[tiab] OR infection control*[tiab] OR patient isolat*[tiab] OR contact precaut*[tiab] OR protective equipment[tiab] OR PPE[tiab] OR protection equipment[tiab] OR isolation of patient*[tiab] OR patient's isolation[tiab] OR professional-patient relations[MeSH Terms] OR patient isolation[MeSH Terms]) AND (impact[tiab] OR attitud*[tiab] OR percept*[tiab] OR experienc*[tiab] OR emotion*[tiab] OR ethic*[tiab] OR empathy[tiab] OR emotions[MeSH Terms] OR empathy[MeSH Terms] OR bioethical issues[MeSH Terms] OR ethics[subheading] OR attitude of health personnel[MeSH Terms] OR health knowledge, attitudes, practice[MeSH Terms] OR social perception[MeSH Terms])

## EMBASE

(healthcare provider*.ti,ab,kf. OR health care provider*.ti,ab,kf. OR health provider*.ti,ab,kf. OR healthcare professional*.ti,ab,kf. OR health care professional*.ti,ab,kf. OR health professional*.ti,ab,kf. OR healthcare worker*.ti,ab,kf. OR health care worker*.ti,ab,kf. OR health worker*.ti,ab,kf. OR healthcare personnel.ti,ab,kf. OR health care personnel.ti,ab,kf. OR health personnel.ti,ab,kf. OR medical personnel.ti,ab,kf. OR healthcare practitioner*.ti,ab,kf. OR health care practitioner*.ti,ab,kf. OR health practitioner*.ti,ab,kf. OR medical staff.ti,ab,kf. OR doctor*.ti,ab,kf. OR physician*.ti,ab,kf. OR clinician*.ti,ab,kf. OR nurs*.ti,ab,kf. OR paramedic*.ti,ab,kf. OR therapist*.ti,ab,kf. OR exp health care personnel/) AND (antibiotic resist*.ti,ab,kf. OR anti-biotic resist*.ti,ab,kf. OR antibiotics resist*.ti,ab,kf. OR anti-biotics resist*.ti,ab,kf. OR antimicrobial drug resist*.ti,ab,kf. OR anti-microbial drug resist*.ti,ab,kf. OR antibiotic drug resist*.ti,ab,kf. OR anti-biotic drug resist*.ti,ab,kf. OR antibacterial drug resist*.ti,ab,kf. OR anti-bacterial drug resist*.ti,ab,kf. OR anti-bacterial resist*.ti,ab,kf. OR antibacterial resist*.ti,ab,kf. OR antibiotic non-suscept*.ti,ab,kf. OR anti-biotic non-suscept*.ti,ab,kf. OR anti-biotic nonsuscept*.ti,ab,kf. OR antibiotic nonsuscept*.ti,ab,kf. OR bacterial drug resist*.ti,ab,kf. OR bacterial resist*.ti,ab,kf. OR bacterium resist*.ti,ab,kf. OR microbial drug resist*.ti,ab,kf. OR aminoglycoside resist*.ti,ab,kf. OR daptomycin resist*.ti,ab,kf. OR fluoroquinolone resist*.ti,ab,kf. OR macrolide resist*.ti,ab,kf. OR polymyxin resist*.ti,ab,kf. OR rifampicin resist*.ti,ab,kf. OR chloramphenicol resist*.ti,ab,kf. OR kanamycin resist*.ti,ab,kf. OR tetracycline resist*.ti,ab,kf. OR trimethoprim resist*.ti,ab,kf. OR vancomycin resist*.ti,ab,kf. OR vancomycine resist*.ti,ab,kf. OR VRE.ti,ab,kf. OR carbapenem resist*.ti,ab,kf. OR CPE.ti,ab,kf. OR extended spectrum beta lactam*.ti,ab,kf. OR extended spectrum betalactam*.ti,ab,kf. OR beta lactam resist*.ti,ab,kf. OR betalactam resist*.ti,ab,kf. OR ESBL.ti,ab,kf. OR MRSA.ti,ab,kf. OR methicillin resist*.ti,ab,kf. OR methicilline resist*.ti,ab,kf. OR multidrug resistant organism*.ti,ab,kf. OR multi drug resistant organism*.ti,ab,kf. OR multiple drug resistant organism*.ti,ab,kf. OR MDRO.ti,ab,kf. OR multidrug resistant microorganism*.ti,ab,kf. OR multidrug resistant micro-organism*.ti,ab,kf. OR multi drug resistant microorganism*.ti,ab,kf. OR multi drug resistant micro-organism*.ti,ab,kf. OR multiple drug resistant microorganism*.ti,ab,kf. OR multiple drug resistant micro-organism*.ti,ab,kf. OR carrier*.ti,ab,kf. OR colonised patient*.ti,ab,kf. OR colonized patient*.ti,ab,kf. OR colonised individ*.ti,ab,kf. OR colonized individ*.ti,ab,kf. OR patient carry*.ti,ab,kf. OR patients carry*.ti,ab,kf. OR patient carri*.ti,ab,kf. OR patients carri*.ti,ab,kf. OR exp antibiotic resistance/) AND (care.ti,ab,kf. OR caring.ti,ab,kf. OR physician-patient interact*.ti,ab,kf. OR patient-physician interact*.ti,ab,kf. OR physician-patient relation*.ti,ab,kf. OR patient-physician relation*.ti,ab,kf. OR doctor-patient interact*.ti,ab,kf. OR patient-doctor interact*.ti,ab,kf. OR doctor-patient relation*.ti,ab,kf. OR patient-doctor relation*.ti,ab,kf. OR nurse-patient interact*.ti,ab,kf. OR patient-nurse interact*.ti,ab,kf. OR nurse-patient relation*.ti,ab,kf. OR patient-nurse relation*.ti,ab,kf. OR professional-patient relation*.ti,ab,kf. OR patient-professional relation*.ti,ab,kf. OR infection control*.ti,ab,kf. OR patient isolat*.ti,ab,kf. OR contact precaut*.ti,ab,kf. OR protective equipment.ti,ab,kf. OR PPE.ti,ab,kf. OR protection equipment.ti,ab,kf. OR isolation of patient*.ti,ab,kf. OR patient's isolation.ti,ab,kf. OR exp professional-patient relationship/ OR exp patient isolation/) AND (impact.ti,ab,kf. OR attitud*.ti,ab,kf. OR percept*.ti,ab,kf. OR experienc*.ti,ab,kf. OR emotion*.ti,ab,kf. OR ethic*.ti,ab,kf. OR empathy.ti,ab,kf. OR emotion/ OR exp bioethics/ OR exp health personnel attitude/ OR attitude to health/)

## PsycINFO

(healthcare provider*.ti,ab,id. OR health care provider*.ti,ab,id. OR health provider*.ti,ab,id. OR healthcare professional*.ti,ab,id. OR health care professional*.ti,ab,id. OR health professional*.ti,ab,id. OR healthcare worker*.ti,ab,id. OR health care worker*.ti,ab,id. OR health worker*.ti,ab,id. OR healthcare personnel.ti,ab,id. OR health care personnel.ti,ab,id. OR health personnel.ti,ab,id. OR medical personnel.ti,ab,id. OR healthcare practitioner*.ti,ab,id. OR health care practitioner*.ti,ab,id. OR health practitioner*.ti,ab,id. OR medical staff.ti,ab,id. OR doctor*.ti,ab,id. OR physician*.ti,ab,id. OR clinician*.ti,ab,id. OR nurs*.ti,ab,id. OR paramedic*.ti,ab,id. OR therapist*.ti,ab,id. OR exp health personnel/) AND (antibiotic resist*.ti,ab,id. OR anti-biotic resist*.ti,ab,id. OR antibiotics resist*.ti,ab,id. OR anti-biotics resist*.ti,ab,id. OR antimicrobial drug resist*.ti,ab,id. OR anti-microbial drug resist*.ti,ab,id. OR antibiotic drug resist*.ti,ab,id. OR anti-biotic drug resist*.ti,ab,id. OR antibacterial drug resist*.ti,ab,id. OR anti-bacterial drug resist*.ti,ab,id. OR anti-bacterial resist*.ti,ab,id. OR antibacterial resist*.ti,ab,id. OR antibiotic non-suscept*.ti,ab,id. OR anti-biotic non-suscept*.ti,ab,id. OR anti-biotic nonsuscept*.ti,ab,id. OR antibiotic nonsuscept*.ti,ab,id. OR bacterial drug resist*.ti,ab,id. OR bacterial resist*.ti,ab,id. OR bacterium resist*.ti,ab,id. OR microbial drug resist*.ti,ab,id. OR aminoglycoside resist*.ti,ab,id. OR daptomycin resist*.ti,ab,id. OR fluoroquinolone resist*.ti,ab,id. OR macrolide resist*.ti,ab,id. OR polymyxin resist*.ti,ab,id. OR rifampicin resist*.ti,ab,id. OR chloramphenicol resist*.ti,ab,id. OR kanamycin resist*.ti,ab,id. OR tetracycline resist*.ti,ab,id. OR trimethoprim resist*.ti,ab,id. OR vancomycin resist*.ti,ab,id. OR vancomycine resist*.ti,ab,id. OR VRE.ti,ab,id. OR carbapenem resist*.ti,ab,id. OR CPE.ti,ab,id. OR extended spectrum beta lactam*.ti,ab,id. OR extended spectrum betalactam*.ti,ab,id. OR beta lactam resist*.ti,ab,id. OR betalactam resist*.ti,ab,id. OR ESBL.ti,ab,id. OR MRSA.ti,ab,id. OR methicillin resist*.ti,ab,id. OR methicilline resist*.ti,ab,id. OR multidrug resistant organism*.ti,ab,id. OR multi drug resistant organism*.ti,ab,id. OR multiple drug resistant organism*.ti,ab,id. OR MDRO.ti,ab,id. OR multidrug resistant microorganism*.ti,ab,id. OR multidrug resistant micro-organism*.ti,ab,id. OR multi drug resistant microorganism*.ti,ab,id. OR multi drug resistant micro-organism*.ti,ab,id. OR multiple drug resistant microorganism*.ti,ab,id. OR multiple drug resistant micro-organism*.ti,ab,id. OR carrier*.ti,ab,id. OR colonised patient*.ti,ab,id. OR colonized patient*.ti,ab,id. OR colonised individ*.ti,ab,id. OR colonized individ*.ti,ab,id. OR patient carry*.ti,ab,id. OR patients carry*.ti,ab,id. OR patient carri*.ti,ab,id. OR patients carri*.ti,ab,id. OR (exp resistance/ and exp antibiotics/)) AND (care.ti,ab,id. OR caring.ti,ab,id. OR physician-patient interact*.ti,ab,id. OR patient-physician interact*.ti,ab,id. OR physician-patient relation*.ti,ab,id. OR patient-physician relation*.ti,ab,id. OR doctor-patient interact*.ti,ab,id. OR patient-doctor interact*.ti,ab,id. OR doctor-patient relation*.ti,ab,id. OR patient-doctor relation*.ti,ab,id. OR nurse-patient interact*.ti,ab,id. OR patient-nurse interact*.ti,ab,id. OR nurse-patient relation*.ti,ab,id. OR patient-nurse relation*.ti,ab,id. OR professional-patient relation*.ti,ab,id. OR patient-professional relation*.ti,ab,id. OR infection control*.ti,ab,id. OR patient isolat*.ti,ab,id. OR contact precaut*.ti,ab,id. OR protective equipment.ti,ab,id. OR PPE.ti,ab,id. OR protection equipment.ti,ab,id. OR isolation of patient*.ti,ab,id. OR patient's isolation.ti,ab,id.) AND (impact.ti,ab,id. OR attitud*.ti,ab,id. OR percept*.ti,ab,id. OR experienc*.ti,ab,id. OR emotion*.ti,ab,id. OR ethic*.ti,ab,id. OR empathy.ti,ab,id. OR empathy/ OR exp bioethics/ OR exp health personnel attitudes/)

## CINAHL

(**TI**("healthcare provider*" OR "health care provider*" OR "health provider*" OR "healthcare professional*" OR "health care professional*" OR "health professional*" OR "healthcare worker*" OR "health care worker*" OR "health worker*" OR "healthcare personnel" OR "health care personnel" OR "health personnel" OR "medical personnel" OR "healthcare practitioner*" OR "health care practitioner*" OR "health practitioner*" OR "medical staff" OR "doctor*" OR "physician*" OR "clinician*" OR "nurs*" OR "paramedic*" OR "therapist*" OR (MH "Health Personnel+") OR **AB**("healthcare provider*" OR "health care provider*" OR "health provider*" OR "healthcare professional*" OR "health care professional*" OR "health professional*" OR "healthcare worker*" OR "health care worker*" OR "health worker*" OR "healthcare personnel" OR "health care personnel" OR "health personnel" OR "medical personnel" OR "healthcare practitioner*" OR "health care practitioner*" OR "health practitioner*" OR "medical staff" OR "doctor*" OR "physician*" OR "clinician*" OR "nurs*" OR "paramedic*" OR "therapist*" OR (MH "Health Personnel+")) AND (**TI**("antibiotic resist*" OR "anti-biotic resist*" OR "antibiotics resist*" OR "anti-biotics resist*" OR "antimicrobial drug resist*" OR "anti-microbial drug resist*" OR "antibiotic drug resist*" OR "anti-biotic drug resist*" OR "antibacterial drug resist*" OR "anti-bacterial drug resist" OR "anti-bacterial resist*" OR "antibacterial resist*" OR "antibiotic non-suscept*" OR "anti-biotic non-suscept*" OR "anti-biotic nonsuscept*" OR "antibiotic nonsuscept*" OR "bacterial drug resist*" OR "bacterial resist*" OR "bacterium resist*" OR "microbial drug resist*" OR "aminoglycoside resist*" OR "daptomycin resist*" OR "fluoroquinolone resist*" OR "macrolide resist*" OR "polymyxin resist*" OR "rifampicin resist*" OR "chloramphenicol resist*" OR "kanamycin resist*" OR "tetracycline resist*" OR "trimethoprim resist*" OR "vancomycin resist*" OR "vancomycine resist*" OR "VRE" OR "carbapenem resist*" OR "CPE" OR "extended spectrum beta lactam*" OR "extended spectrum betalactam*" OR "beta lactam resist*" OR "betalactam resist*" OR "ESBL" OR "MRSA" OR "methicillin resist*" OR "methicilline resist*" OR "multidrug resistant organism*" OR "multi drug resistant organism*" OR "multiple drug resistant organism*" OR "MDRO" OR "multidrug resistant microorganism*" OR "multidrug resistant micro-organism*" OR "multi drug resistant microorganism*" OR "multi drug resistant micro-organism*" OR "multiple drug resistant microorganism*" OR "multiple drug resistant micro-organism*" OR "carrier*" OR "colonised patient*" OR "colonized patient*" OR "colonised individ*" OR "colonized individ*" OR "patient carry*" OR "patients carry*" OR "patient carri*" OR "patients carri*" OR (MH "Carrier State") OR (MH "Drug Resistance, Microbial+") OR **AB**("antibiotic resist*" OR "anti-biotic resist*" OR "antibiotics resist*" OR "anti-biotics resist*" OR "antimicrobial drug resist*" OR "anti-microbial drug resist*" OR "antibiotic drug resist*" OR "anti-biotic drug resist*" OR "antibacterial drug resist*" OR "anti-bacterial drug resist" OR "anti-bacterial resist*" OR "antibacterial resist*" OR "antibiotic non-suscept*" OR "anti-biotic non-suscept*" OR "anti-biotic nonsuscept*" OR "antibiotic nonsuscept*" OR "bacterial drug resist*" OR "bacterial resist*" OR "bacterium resist*" OR "microbial drug resist*" OR "aminoglycoside resist*" OR "daptomycin resist*" OR "fluoroquinolone resist*" OR "macrolide resist*" OR "polymyxin resist*" OR "rifampicin resist*" OR "chloramphenicol resist*" OR "kanamycin resist*" OR "tetracycline resist*" OR "trimethoprim resist*" OR "vancomycin resist*" OR "vancomycine resist*" OR "VRE" OR "carbapenem resist*" OR "CPE" OR "extended spectrum beta lactam*" OR "extended spectrum betalactam*" OR "beta lactam resist*" OR "betalactam resist*" OR "ESBL" OR "MRSA" OR "methicillin resist*" OR "methicilline resist*" OR "multidrug resistant organism*" OR "multi drug resistant organism*" OR "multiple drug resistant organism*" OR "MDRO" OR "multidrug resistant microorganism*" OR "multidrug resistant micro-organism*" OR "multi drug resistant microorganism*" OR "multi drug resistant micro-organism*" OR "multiple drug resistant microorganism*" OR "multiple drug resistant micro-organism*" OR "carrier*" OR "colonised patient*" OR "colonized patient*" OR "colonised individ*" OR "colonized individ*" OR "patient carry*" OR "patients carry*" OR "patient carri*" OR "patients carri*" OR (MH "Carrier State") OR (MH "Drug Resistance, Microbial+")) AND (**TI**("care" OR "caring" OR "physician-patient interact*" OR "patient-physician interact*" OR "physician-patient relation*" OR "patient-physician relation*" OR "doctor-patient interact*" OR "patient-doctor interact*" OR "doctor-patient relation*" OR "patient-doctor relation*" OR "nurse-patient interact*" OR "patient-nurse interact*" OR "nurse-patient relation*" OR "patient-nurse relation*" OR "professional-patient relation*" OR "patient-professional relation*" OR "infection control*" OR "patient isolat*" OR "contact precaut*" OR "protective equipment" OR PPE OR "protection equipment" OR "isolation of patient*" OR "patient's isolation" OR "contact and isolation precaut*" OR (MH "professional-patient relations"+) OR (MH "Patient Isolation") OR **AB**("care" OR "caring" OR "physician-patient interact*" OR "patient-physician interact*" OR "physician-patient relation*" OR "patient-physician relation*" OR "doctor-patient interact*" OR "patient-doctor interact*" OR "doctor-patient relation*" OR "patient-doctor relation*" OR "nurse-patient interact*" OR "patient-nurse interact*" OR "nurse-patient relation*" OR "patient-nurse relation*" OR "professional-patient relation*" OR "patient-professional relation*" OR "infection control*" OR "patient isolat*" OR "contact precaut*" OR "protective equipment" OR PPE OR "protection equipment" OR "isolation of patient*" OR "patient's isolation" OR (MH "professional-patient relations"+) OR (MH "Patient Isolation")) AND (**TI**("impact" OR "attitud*" OR "percept*" OR "experienc*" OR "emotion*" OR "ethic*" OR "empathy" OR (MH "Emotions+") OR (MH "Empathy") OR (MH "bioethics") OR (MH "attitude of health personnel+") OR (MH "social perception+") OR **AB**("impact" OR "attitud*" OR "percept*" OR "experienc*" OR "emotion*" OR "ethic*" OR "empathy" OR (MH "Emotions+") OR (MH "Empathy") OR (MH "bioethics") OR (MH "attitude of health personnel+") OR (MH "social perception+"))

## Web of Science

(TS=("healthcare provider*" OR "health care provider*" OR "health provider*" OR "healthcare professional*" OR "health care professional*" OR "health professional*" OR "healthcare worker*" OR "health care worker*" OR "health worker*" OR "healthcare personnel" OR "health care personnel" OR "health personnel" OR "medical personnel" OR "healthcare practitioner*" OR "health care practitioner*" OR "health practitioner*" OR "medical staff" OR "doctor*" OR "physician*" OR "clinician*" OR "nurs*" OR "paramedic*" OR "therapist*")) AND (TS=("antibiotic resist*" OR "anti-biotic resist*" OR "antibiotics resist*" OR "anti-biotics resist*" OR "antimicrobial drug resist*" OR "anti-microbial drug resist*" OR "antibiotic drug resist*" OR "anti-biotic drug resist*" OR "antibacterial drug resist*" OR "anti-bacterial drug resist" OR "anti-bacterial resist*" OR "antibacterial resist*" OR "antibiotic non-suscept*" OR "anti-biotic non-suscept*" OR "anti-biotic nonsuscept*" OR "antibiotic nonsuscept*" OR "bacterial drug resist*" OR "bacterial resist*" OR "bacterium resist*" OR "microbial drug resist*" OR "aminoglycoside resist*" OR "daptomycin resist*" OR "fluoroquinolone resist*" OR "macrolide resist*" OR "polymyxin resist*" OR "rifampicin resist*" OR "chloramphenicol resist*" OR "kanamycin resist*" OR "tetracycline resist*" OR "trimethoprim resist*" OR "vancomycin resist*" OR "vancomycine resist*" OR "VRE" OR "carbapenem resist*" OR "CPE" OR "extended spectrum beta lactam*" OR "extended spectrum betalactam*" OR "beta lactam resist*" OR "betalactam resist*" OR "ESBL" OR "MRSA" OR "methicillin resist*" OR "methicilline resist*" OR "multidrug resistant organism*" OR "multi drug resistant organism*" OR "multiple drug resistant organism*" OR "MDRO" OR "multidrug resistant microorganism*" OR "multidrug resistant micro-organism*" OR "multi drug resistant microorganism*" OR "multi drug resistant micro-organism*" OR "multiple drug resistant microorganism*" OR "multiple drug resistant micro-organism*" OR "carrier*" OR "colonised patient*" OR "colonized patient*" OR "colonised individ*" OR "colonized individ*" OR "patient carry*" OR "patients carry*" OR "patient carri*" OR "patients carri*")) AND (TS=("care" OR "caring" OR "physician-patient interact*" OR "patient-physician interact*" OR "physician-patient relation*" OR "patient-physician relation*" OR "doctor-patient interact*" OR "patient-doctor interact*" OR "doctor-patient relation*" OR "patient-doctor relation*" OR "nurse-patient interact*" OR "patient-nurse interact*" OR "nurse-patient relation*" OR "patient-nurse relation*" OR "professional-patient relation*" OR "patient-professional relation*" OR "infection control*" OR "patient isolat*" OR "contact precaut*" OR "protective equipment" OR "PPE" OR "protection equipment" OR "isolation of patient*" OR "patient's isolation")) AND (TS=("impact" OR "attitud*" OR "percept*" OR "experienc*" OR "emotion*" OR "ethic*" OR "empathy"))

## Cochrane library

("healthcare" NEXT provider*):ti,ab,kw OR ("health care" NEXT provider*):ti,ab,kw OR ("health" NEXT provider*):ti,ab,kw OR ("healthcare" NEXT professional*):ti,ab,kw OR ("health care" NEXT professional*):ti,ab,kw OR ("health" NEXT professional*):ti,ab,kw OR ("healthcare" NEXT worker*):ti,ab,kw OR ("health care" NEXT worker*):ti,ab,kw OR ("health" NEXT worker*):ti,ab,kw OR "healthcare personnel":ti,ab,kw OR "health care personnel":ti,ab,kw OR "health personnel":ti,ab,kw OR "medical personnel":ti,ab,kw OR ("healthcare" NEXT practitioner*):ti,ab,kw OR ("health care" NEXT practitioner*):ti,ab,kw OR ("health" NEXT practitioner*):ti,ab,kw OR "medical staff":ti,ab,kw OR doctor*:ti,ab,kw OR physician*:ti,ab,kw OR clinician*:ti,ab,kw OR nurs*:ti,ab,kw OR paramedic*:ti,ab,kw OR therapist*:ti,ab,kw OR [mh "health personnel"] AND ("antibiotic" NEXT resist*):ti,ab,kw OR ("anti-biotic" NEXT resist*):ti,ab,kw OR ("antibiotics" NEXT resist*):ti,ab,kw OR ("anti-biotics" NEXT resist*):ti,ab,kw OR ("antimicrobial drug" NEXT resist*):ti,ab,kw OR ("anti-microbial drug" NEXT resist*):ti,ab,kw OR ("antibiotic drug" NEXT resist*):ti,ab,kw OR ("anti-biotic drug" NEXT resist*):ti,ab,kw OR ("antibacterial drug" NEXT resist*):ti,ab,kw OR ("anti-bacterial drug" NEXT resist*):ti,ab,kw OR ("anti-bacterial" NEXT resist*):ti,ab,kw OR ("antibacterial" NEXT resist*):ti,ab,kw OR ("antibiotic" NEXT non-suscept*):ti,ab,kw OR ("antibiotic" NEXT nonsuscept*):ti,ab,kw OR ("anti-biotic" NEXT non-suscept*):ti,ab,kw OR ("anti-biotic" NEXT nonsuscept*):ti,ab,kw OR ("bacterial drug" NEXT resist*):ti,ab,kw OR ("bacterial" NEXT resist*):ti,ab,kw OR ("bacterium" NEXT resist*):ti,ab,kw OR ("microbial drug" NEXT resist*):ti,ab,kw OR ("aminoglycoside" NEXT resist*):ti,ab,kw OR ("daptomycin" NEXT resist*):ti,ab,kw OR ("fluoroquinolone" NEXT resist*):ti,ab,kw OR ("macrolide" NEXT resist*):ti,ab,kw OR ("polymyxin" NEXT resist*):ti,ab,kw OR ("rifampicin" NEXT resist*):ti,ab,kw OR ("chloramphenicol" NEXT resist*):ti,ab,kw OR ("kanamycin" NEXT resist*):ti,ab,kw OR ("tetracycline" NEXT resist*):ti,ab,kw OR ("trimethoprim" NEXT resist*):ti,ab,kw OR ("vancomycin" NEXT resist*):ti,ab,kw OR ("vancomycine" NEXT resist*):ti,ab,kw OR VRE:ti,ab,kw OR ("carbapenem" NEXT resist*):ti,ab,kw OR CPE:ti,ab,kw OR ("extended spectrum beta" NEXT lactam*):ti,ab,kw OR ("extended spectrum" NEXT betalactam*):ti,ab,kw OR ("beta lactam" NEXT resist*):ti,ab,kw OR ("betalactam" NEXT resist*):ti,ab,kw OR ESBL:ti,ab,kw OR MRSA:ti,ab,kw OR ("methicillin" NEXT resist*):ti,ab,kw OR ("methicilline" NEXT resist*):ti,ab,kw OR ("multidrug resistant" NEXT organism*):ti,ab,kw OR ("multi drug resistant" NEXT organism*):ti,ab,kw OR ("multiple drug resistant" NEXT organism*):ti,ab,kw OR MDRO:ti,ab,kw OR ("multidrug resistant" NEXT microorganism*):ti,ab,kw OR ("multidrug resistant" NEXT micro-organism*):ti,ab,kw OR ("multi drug resistant" NEXT microorganism*):ti,ab,kw OR ("multi drug resistant" NEXT micro-organism*):ti,ab,kw OR ("multiple drug resistant" NEXT microorganism):ti,ab,kw OR ("multiple drug resistant" NEXT micro-organism*):ti,ab,kw OR carrier*:ti,ab,kw OR ("colonised" NEXT patient*):ti,ab,kw OR ("colonized" NEXT patient*):ti,ab,kw OR ("colonised" NEXT individ*):ti,ab,kw OR ("colonized" NEXT individ*):ti,ab,kw OR ("patient" NEXT carry*):ti,ab,kw OR ("patients" NEXT carry*):ti,ab,kw OR ("patient" NEXT carri*):ti,ab,kw OR ("patients" NEXT carri*):ti,ab,kw OR [mh "carrier state"] OR [mh "drug resistance, microbial"] AND (care:ti,ab,kw OR caring:ti,ab,kw OR ("physician-patient" NEXT interact*):ti,ab,kw OR ("patient-physician" NEXT interact*):ti,ab,kw OR ("physician-patient" NEXT relation*):ti,ab,kw OR ("patient-physician" NEXT relation*):ti,ab,kw OR ("doctor-patient" NEXT interact*):ti,ab,kw OR ("patient-doctor" NEXT interact*):ti,ab,kw OR ("doctor-patient" NEXT relation*):ti,ab,kw OR ("patient-doctor" NEXT relation*):ti,ab,kw OR ("nurse-patient" NEXT interact*):ti,ab,kw OR ("patient-nurse" NEXT interact*):ti,ab,kw OR ("nurse-patient" NEXT relation*):ti,ab,kw OR ("patient-nurse" NEXT relation*):ti,ab,kw OR ("professional-patient" NEXT relation*):ti,ab,kw OR ("patient-professional" NEXT relation*):ti,ab,kw OR ("infection" NEXT control*):ti,ab,kw OR ("patient" NEXT isolat*):ti,ab,kw OR ("contact" NEXT precaut*):ti,ab,kw OR "protective equipment":ti,ab,kw OR PPE:ti,ab,kw OR "protection equipment":ti,ab,kw OR ("isolation of" NEXT patient*):ti,ab,kw OR "patient's isolation":ti,ab,kw OR [mh "professional-patient relations"] OR [mh "patient isolation"] AND (impact:ti,ab,kw OR attitud*:ti,ab,kw OR percept*:ti,ab,kw OR experienc*:ti,ab,kw OR emotion*:ti,ab,kw OR ethic*:ti,ab,kw OR empathy:ti,ab,kw OR [mh emotions] OR [mh empathy] OR [mh "bioethical issues"] OR [mh "attitude of health personnel"] OR [mh "health knowledge, attitudes, practice"] OR [mh "social perception"]

**Table S3: Eligibility criteria**

## inclusion criteria

- Peer-reviewed publication
- Written in English/Dutch
- Study contains original data
- Study provides thoughts, feelings (and eventually how this impacts actions) of HCPs
- Study reports from the perspective of HCPs
- Study concerns resistant bacteria

## exclusion criteria

- Published as book chapters, dissertations, reviews, grey literature, background articles, editorials, commentaries, abstracts
- Ineligible languages
- Study reports on impact of being a carrier as a healthcare provider
- Study provides no insight into experiences, such as frequency of visits only
- Study concerns resistant micro-organisms which are not bacteria
- Full text is not available after contacting the corresponding author twice

**Table S4:** **Quality assessed using the mixed methods appraisal tool (MMAT) 2018**

According to: Hong QN, Fàbregues S, Bartlett G *et al.* The Mixed Methods Appraisal Tool (MMAT) version 2018 for information professionals and researchers. Education for information. 2018;34(4):285-291.

| **Screening questions** | |
| --- | --- |
| **S1** | Are there clear research questions? |
| **S2** | Do the collected data allow to address the research questions? |
|  |  |
| **1. Qualitative** | |
| **1.1** | Is the qualitative approach appropriate to answer the research question? |
| **1.2** | Are the qualitative data collection methods adequate to address the research question? |
| **1.3** | Are the findings adequately derived from the data? |
| **1.4** | Is the interpretation of results sufficiently substantiated by data? |
| **1.5** | Is there coherence between qualitative data sources, collection, analysis and interpretation? |
|  |  |
| **4. Quantitative descriptive** | |
| **4.1** | Is the sampling strategy relevant to address the research question? |
| **4.2** | Is the sample representative of the target population? |
| **4.3** | Are the measurements appropriate? |
| **4.4** | Is the risk of nonresponse bias low? |
| **4.5** | Is the statistical analysis appropriate to answer the research question? |

| **Article** | **Category of study designs** | **Screening questions** | | **1. Qualitative** | | | | | **4. Quantitative descriptive** | | | | |
| --- | --- | --- | --- | --- | --- | --- | --- | --- | --- | --- | --- | --- | --- |
|  |  | **S1.** | **S2.** | **1.1** | **1.2** | **1.3** | **1.4** | **1.5** | **4.1** | **4.2** | **4.3** | **4.4** | **4.5** |
| Andersson 2016 | Qualitative studies | Y | Y | Y | Y | Y | CT | Y |  |  |  |  |  |
| DaSilva 2010 | Quantitative descriptive studies | Y | Y |  |  |  |  |  | CT | Y | CT | CT | Y |
| Eli 2020 | Qualitative studies | Y | Y | Y | Y | Y | Y | Y |  |  |  |  |  |
| Harris 2020 | Quantitative descriptive studies | Y | Y |  |  |  |  |  | Y | CT | Y | Y | N/A |
| Harris 2023 | Qualitative studies | Y | Y | Y | Y | Y | Y | Y |  |  |  |  |  |
| Herbst 2019 | Qualitative studies | Y | Y | Y | Y | CT | Y | Y |  |  |  |  |  |
| Kaba 2017 | Qualitative studies | Y | Y | Y | Y | CT | CT | CT |  |  |  |  |  |
| Khan 2006 | Quantitative descriptive studies | Y | Y |  |  |  |  |  | Y | Y | Y | CT | N |
| Krein 2020 | Quantitative descriptive studies | Y | Y |  |  |  |  |  | Y | CT | CT | CT | CT |
| Langeveld 2022 | Quantitative descriptive studies | Y | Y |  |  |  |  |  | Y | Y | Y | Y | Y |
| Langeveld 2024 | Qualitative studies | Y | Y | Y | Y | Y | Y | Y |  |  |  |  |  |
| Lindberg 2014 | Qualitative studies | Y | Y | Y | Y | Y | N | Y |  |  |  |  |  |
| Mitchell 2002 | Qualitative studies | Y | Y | Y | CT | CT | Y | Y |  |  |  |  |  |
| O’Connor 2023 | Qualitative studies | Y | Y | Y | Y | Y | Y | Y |  |  |  |  |  |
| Seibert 2014 | Qualitative studies | Y | Y | Y | Y | Y | Y | Y |  |  |  |  |  |
| Tiedtke 2018 | Qualitative studies | Y | Y | Y | Y | Y | Y | Y |  |  |  |  |  |
| Watson 2023 | Quantitative descriptive studies | Y | Y |  |  |  |  |  | Y | Y | Y | CT | Y |
| Wiklund 2015 | Qualitative studies | Y | Y | Y | Y | Y | Y | Y |  |  |  |  |  |

*Legend:* Y=Yes, CT=Cannot tell (i.e., not present, thus no possibility to judge), N=No, N/A=not applicable for this study.

**Table S5: Excluded studies**

| **Reason for exclusion** |  |
| --- | --- |
| Study is not about the personal perspective of healthcare providers | Bushuven S, Dietz A, Bushuven S et al. Interprofessional perceptions and emotional impact of multidrug-resistant organisms: A qualitative study. *American Journal of Infection Control* 2019; **47**: 876-82.  Masse V, Valiquette L, Boukhoudmi S et al. Impact of Methicillin Resistant Staphylococcus aureus Contact Isolation Units on Medical Care. *PLOS ONE* 2013; **8**: e57057.  Mutsonziwa GA, Green J, Blundell J. Registered nurses’ perspectives on how patients with multi-drug resistant organisms experience isolation. *Infection, Disease & Health* 2021; **26**: 22-30. |
| Study provides no insight into experiences (such as frequency of visits only) | van Dulm E, van der Veldt W, der Meiden KJ-v et al. Perceived barriers and enablers for preventing the spread of carbapenem producing gram-negative bacteria during patient transfers: a mixed methods study among healthcare providers. *BMC Infectious Diseases* 2019; **19**: 1050.  Easton PM, Sarma A, Williams FL et al. Infection control and management of MRSA: assessing the knowledge of staff in an acute hospital setting. *J Hosp Infect* 2007; **66**: 29-33.  Furuno JP, Krein S, Lansing B et al. Health care worker opinions on use of isolation precautions in long-term care facilities. *American Journal of Infection Control* 2012; **40**: 263-6.  Lugg GR, Ahmed HA. Nurses' perceptions of meticillin-resistant Staphylococcus aureus: Impacts on practice. *British Journal of Infection Control* 2008; **9**: 8-14.  Marschall P, Hübner N-O, Maletzki S et al. Attitudes and perceptions of health care workers in Northeastern Germany about multidrug-resistant organisms. *American journal of infection control* 2016; **44**: e91-e4.  McClean P, Tunney M, Parsons C et al. Infection control and meticillin-resistant Staphylococcus aureus decolonization: the perspective of nursing home staff. *Journal of Hospital Infection* 2012; **81**: 264-9.  Pant ND, Sharma M. Carriage of methicillin resistant Staphylococcus aureus and awareness of infection control among health care workers working in intensive care unit of a hospital in Nepal. *Braz J Infect Dis* 2016; **20**: 218-9.  Pineles L, Petruccelli C, Perencevich EN et al. The Impact of Isolation on Healthcare Worker Contact and Compliance With Infection Control Practices in Nursing Homes. *Infect Control Hosp Epidemiol* 2018; **39**: 683-7.  Raupach-Rosin H, Rübsamen N, Szkopek S et al. Care for MRSA carriers in the outpatient sector: a survey among MRSA carriers and physicians in two regions in Germany. *BMC Infectious Diseases* 2016; **16**: 184.  Rynkiewich KR, Makhija J, Froilan MCM et al. Healthcare personnel experiences implementing carbapenem-resistant Enterobacterales infection control measures at a ventilator-capable skilled nursing facility—A qualitative analysis. *Infection Control & Hospital Epidemiology* 2022; **43**: 1010-6.  Saint S, Higgins LA, Nallamothu BK et al. Do physicians examine patients in contact isolation less frequently? A brief report. *American Journal of Infection Control* 2003; **31**: 354-6.  Schmidt P, Hartenstein-Pinter A, Wager J et al. Addressing multidrug resistant pathogens in pediatric palliative care patients—the nurses point of view: A qualitative study. *Palliative Medicine* 2020; **34**: 349-57.  Vaillant L, Birgand G, Esposito-Farese M et al. Awareness among French healthcare workers of the transmission of multidrug resistant organisms: a large cross-sectional survey. *Antimicrobial Resistance & Infection Control* 2019; **8**: 173.  Wolf R, Lewis D, Cochran R et al. Nursing staff perceptions of methicillin-resistant Staphylococcus aureus and infection control in a long-term care facility. *J Am Med Dir Assoc* 2008; **9**: 342-6.  Zimmerman P-A, Rowe J, Wallis M. Accommodating patients with a history of colonisation or infection with a multi-resistant organism: a case study investigation. *Australian Infection Control* 2004; **9**: 30-5. |
| Study is not about carriership | Arjun SD, Matlakala MC, Mavundla TR. Enrolled nurses' experiences of caring for multi-drug resistant tuberculosis patients in the Kwa-Zulu Natal Province of South Africa. *Africa Journal of Nursing and Midwifery* 2023; **15**: 54-67.  Giblin TB, Sinkowitz-Cochran RL, Harris PL et al. Clinicians' perceptions of the problem of antimicrobial resistance in health care facilities. *Arch Intern Med* 2004; **164**: 1662-8. |
| Full text is not available after contacting the corresponding author twice | Hurri S. Experiences of influence of MRSA infection control in work and working community in long-term care institutions. *Sairaanhoitaja* 2006; **79**: 5-11.  Novakova I, Neveur M. Managing multi-drug resistant bacteria in nursing homes. *Revue de l'Infirmiere* 2013: 20-3. |
| Study does not include original data | Malhotra A, Chaudhuri P, Chahal A. Impact of Quarantine on Psychological and Psychosocial Aspects in Individual and Health Care Staff. *Medico Legal Update* 2020; **20**: 1540-4.  Szczypta A, Talaga K, Bulanda M. Nursing care for patients infected or colonized with vancomycin-resistant enterococci (VRE). *Folia Med Cracov* 2016; **56**: 13-20. |
